# Estimation of Physical Activity Energy Expenditure during Free-Living from Wrist Accelerometry in UK Adults

**DOI:** 10.1371/journal.pone.0167472

**Published:** 2016-12-09

**Authors:** Tom White, Kate Westgate, Nicholas J. Wareham, Soren Brage

**Affiliations:** MRC Epidemiology Unit, University of Cambridge, Cambridge, United Kingdom; West Virginia University, UNITED STATES

## Abstract

**Background:**

Wrist-worn accelerometers are emerging as the most common instrument for measuring physical activity in large-scale epidemiological studies, though little is known about the relationship between wrist acceleration and physical activity energy expenditure (PAEE).

**Methods:**

1695 UK adults wore two devices simultaneously for six days; a combined sensor and a wrist accelerometer. The combined sensor measured heart rate and trunk acceleration, which was combined with a treadmill test to yield a signal of individually-calibrated PAEE. Multi-level regression models were used to characterise the relationship between the two time-series, and their estimations were evaluated in an independent holdout sample. Finally, the relationship between PAEE and BMI was described separately for each source of PAEE estimate (wrist acceleration models and combined-sensing).

**Results:**

Wrist acceleration explained 44–47% between-individual variance in PAEE, with RMSE between 34–39 J•min^-1^•kg^-1^. Estimations agreed well with PAEE in cross-validation (mean bias [95% limits of agreement]: 0.07 [-70.6:70.7]) but overestimated in women by 3% and underestimated in men by 4%. Estimation error was inversely related to age (-2.3 J•min^-1^•kg^-1^ per 10y) and BMI (-0.3 J•min^-1^•kg^-1^ per kg/m^2^). Associations with BMI were similar for all PAEE estimates (approximately -0.08 kg/m^2^ per J•min^-1^•kg^-1^).

**Conclusions:**

A strong relationship exists between wrist acceleration and PAEE in free-living adults, such that irrespective of the objective method of PAEE assessment, a strong inverse association between PAEE and BMI was observed.

## Introduction

Physical activity (PA) is important for the prevention of several chronic diseases such as diabetes, cardiovascular disease, and certain cancers [1]. However, there is uncertainty about the dose-response relationships as well as the prevalence of the exposure, owing to difficulty in assessing habitual physical activity accurately [2]. Several methods now exist but wrist accelerometry is becoming a more common objective measure of habitual physical activity in large-scale epidemiological studies [3,4], due to its relative low cost and high acceptability to study participants. This necessitates a better understanding of the relationship between wrist acceleration and other measures of physical activity so that estimates of prevalence and disease relationships can be compared between populations assessed using different methods.

A recent consensus statement expressed the imminent need for harmonisation of accelerometry data collected in free-living adults [5]. The current lack of comparability between measurement modalities limits possibilities of assessing the global prevalence of physical activity, or pooling data from multiple sources to better understand its relationship with disease. For example, a meta-analysis aiming to determine whether physical activity attenuates the effect of the FTO gene on obesity risk was forced to dichotomise physical activity (active or inactive) across the multitude of exposure measures [6]; while this was sufficient to confirm the existence of an interaction, it was not possible to determine what dose of activity was necessary to protect against the deleterious FTO variant.

An important component of physical activity is its associated increase in energy expenditure (PAEE). If PAEE is captured during free-living in high time resolution, this produces intensity time-series data that can be used to describe a person’s behavioural profile. A number of previous studies have validated wrist acceleration derivatives against gold-standard measures of energy expenditure, such as the doubly-labelled water (DLW) method[7] and indirect calorimetry from respiratory gas analysis [8]. However, the high cost of DLW has prohibited such work in large population samples, and the nature of the measurement only allows the exploration of total activity volume, rather than the underlying intensity time-series. Breath-by-breath analysis does provide intensity time-series data but the method is not a feasible solution for monitoring energy expenditure in free-living. While laboratory-based comparisons have utility in elucidating the relationships between wrist acceleration and energy expenditure during specific activities, such experiments are unlikely to adequately capture the full spectrum of human activities in representative proportions, and we remain ill-equipped to recognise different activity types in free-living records.

The purpose of this study was to complement existing validation studies by building predictive models of classic PA measures from wrist acceleration derivatives, using both acceleration of the trunk and PAEE collected *in free-living* as criteria. We then evaluate the derived models by cross-validation in age, sex, and BMI strata, and finally investigate if model performance translates into valid methods of harmonisation by examining their association with obesity, compared to that of the criterion measure.

## Methods

This dataset was part of the Fenland Study [9], an ongoing prospective cohort study designed to identify the behavioural, environmental and genetic causes of obesity and type 2 diabetes. Participants were recruited to attend one of three clinical research facilities in the region surrounding Cambridge, UK. All participants provided written informed consent and the study was approved by the local ethics committee (NRES Committee–East of England Cambridge Central) and performed in accordance with the Declaration of Helsinki.

A subsample of 1695 participants were asked to wear two devices simultaneously; a combined heart rate and movement sensor (Actiheart, CamNtech, Cambridgeshire, UK), which measured heart rate and uniaxial acceleration of the trunk in 15-second intervals [10], and a wrist accelerometer (GeneActiv, ActivInsights, Cambridgeshire, UK) worn on the non-dominant wrist, which recorded triaxial acceleration at 60 Hertz. Participants were asked to wear the monitors for 6 complete days and advised that both monitors were waterproof and could be worn continuously including during showering and sleeping.

At the clinic visit, prior to the free-living monitoring period, participants performed a ramped treadmill test to establish their individual heart rate response to a submaximal test [11]. These measurements produced calibration parameters to inform a branched equation model of PAEE [12], which has been validated against instantaneous PAEE (intensity) from indirect calorimetry [13,14]. Following pre-processing of the heart rate data collected during free-living to eliminate potential noise [15], the branched equation model was applied to calculate instantaneous PAEE (J•min^-1^•kg^-1^). This methodology has been successfully validated against PAEE from DLW in several populations [16,17], including a sample of UK men and women where it was shown to explain 41% of the variance in free-living PAEE and with no mean bias [18].

The raw triaxial wrist acceleration data was auto-calibrated to local gravitational acceleration (in *g*) using a method described elsewhere [19]. The calibrated acceleration was then used to calculate Vector Magnitude (VM) per sample: $VM\;(X,Y,Z)=\sqrt{X^{2}+Y^{2}+Z^{2}}$

VM, or Euclidean Norm, can be interpreted as the magnitude of acceleration the device was subjected to at each measurement, including gravitational acceleration. There will also be a potential sensor noise component in the high frequency domain (above human physiological range), which we filtered out by a 20 Hertz low-pass filter. In the present study, we calculated two derivatives of VM, both aiming to remove the gravity component from the signal in order to isolate the activity-related acceleration component; 1) Euclidean Norm Minus One (ENMO) subtracts 1g from VM and truncates the result to zero at sample level, whereas 2) High-Pass Filtered Vector Magnitude (HPFVM) applies a high-pass filter to the VM signal at 0.2 Hertz, therefore treating gravity as a low-frequency component to be filtered out. These two signals, ENMO and HPFVM, are both plausible approximations of acceleration as a result of human movement [20], and are the primary descriptions of wrist acceleration used in the following analyses.

Non-wear detection procedures were applied to both the wrist acceleration [7] and combined-sensing traces [18], and any such non-wear periods were excluded from these analyses. Briefly, non-wear in the wrist acceleration data was defined as time periods where the standard deviation of acceleration in each axis fell below 13*mg* for longer than 1 hour, and non-wear in the combined sensing data was defined as extended periods of non-physiological heart rate concomitantly with extended (>90min) periods of zero movement registered by the accelerometer.

**Fig 1:**
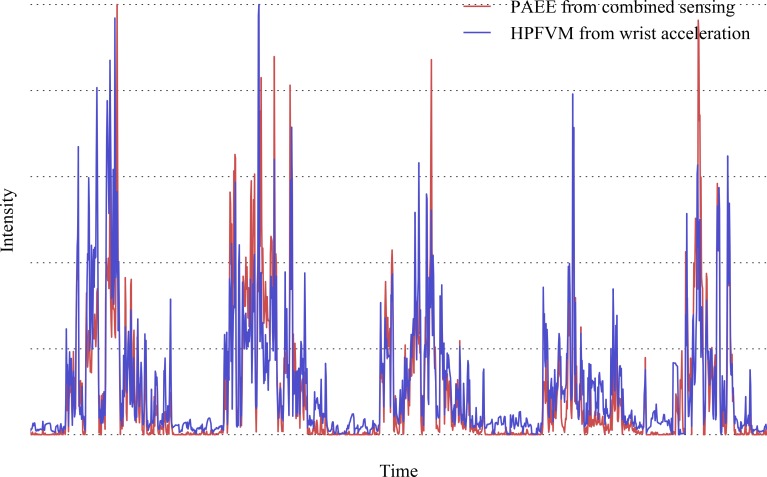
Example of simultaneous PAEE and wrist acceleration signal over 5 days.

All signals were summarised to a common time resolution of one observation per 5 minutes, an example of which is shown in Fig 1. This was chosen as an appropriate window length based on a variety of competing considerations. Firstly, the time-lagged physiological response of heart rate to movement precludes an instantaneous comparison and necessitates a physiologically appropriate time buffer. Secondly, due to hardware limitations and initialisation conditions, we could not guarantee a *perfect* time synchronisation between the two monitors. Finally, maintaining the highest possible time resolution within these constraints preserves the most variance in the intensity time-series, and maximises the number of observations in the dataset. The models derived in this work (described in detail below) exclusively use time-invariant signal features such as arithmetic means; this means that they are robust to changes in window size, and it is therefore equally appropriate to use them to estimate hourly or daily outputs from hourly or daily inputs.

Multi-level linear regression models were designed to independently predict PAEE and trunk acceleration from wrist acceleration. Four models were tested: a linear and quadratic model for each of ENMO and HPFVM.

1. $\alpha +\beta_{1}(ENMO)$
2. $\alpha +\beta_{1}(ENMO)+\beta_{2}(\sqrt{ENMO})+\beta_{3}(ENMO^{2})$
3. $\alpha +\beta_{1}(HPFVM)$
4. $\alpha +\beta_{1}(HPFVM)+\beta_{2}(\sqrt{HPFVM})+\beta_{3}(HPFVM^{2})$

The models were derived in a randomly chosen subset containing 60% of *people* in the cohort (n = 1050) and evaluated in the remaining 40% (n = 645). Model performance was assessed using within- and between-individual explained variances (Pearson’s coefficient) and Root Mean Squared Error (RMSE) metrics, as determined from ANOVA repeated measures modelling specified with random effects at the participant level. After assessing the performance of these models on the test dataset as a whole, we selected the strongest model and tested for differential bias by sex, age and BMI categories of under/normal-weight, overweight, and obese (<25, ≥25 & <30, ≥30 kg/m^2^, respectively) within the test dataset. All statistical tests were performed in Stata version 14 (StataCorp, Texas, USA).

In order to test the epidemiological utility of the derived models, we examined the associations between BMI and PAEE in the test dataset (n = 645). Using our criterion PAEE measure, we first characterised the linear dose-response relationship with BMI, adjusting for age and sex. We then repeated this analysis using *predicted* PAEE from each of the derived prediction models, and compared the beta coefficients and 95% confidence intervals to those using criterion PAEE.

## Results

A description of the population sample included in this analysis is given in Table 1. In total, 1752287 valid 5-minute windows from 1050 individuals were included in the training dataset; the median number of observations per individual was 1738, equating to just over 6 days. Mean PAEE across the sample was 36.4 J•min^-1^•kg^-1^, with higher average in men than women (38.1 and 34.7, respectively). Mean wrist acceleration according to both the ENMO and the HPFVM metrics was similar in men and women.

**Table 1:** Summary statistics of the cohort, provided separately for the training and test datasets, by sex.

	Train		Test	
	Men	Women	Men	Women
N	499	551	305	340
Age (years)	49.68 (7.29)	50.07 (7.00)	51.58 (6.97)	49.90 (7.29)
Height (m)	1.78 (0.06)	1.63 (0.06)	1.77 (0.06)	1.64 (0.06)
Weight (kg)	85.85 (14.02)	69.97 (13.05)	85.50 (13.01)	69.44 (12.85)
BMI (kg•m^-2^)	26.98 (4.08)	26.05 (4.86)	26.99 (3.90)	25.68 (4.71)
PAEE (J•min^-1^•kg^-1^)	39.10 (16.44)	34.51 (13.50)	37.41 (15.57)	35.54 (14.46)
Trunk ACC (m•s^-2^)	0.12 (0.05)	0.13 (0.06)	0.12 (0.05)	0.13 (0.05)
Wrist ACC, ENMO (mg)	32.15 (9.28)	31.25 (8.12)	31.36 (9.17)	31.59 (7.92)
Wrist ACC, HPFVM (mg)	49.17 (11.73)	47.75 (10.35)	47.89 (11.81)	48.09 (10.23)

Values given are mean (standard deviation).

**Fig 2:**
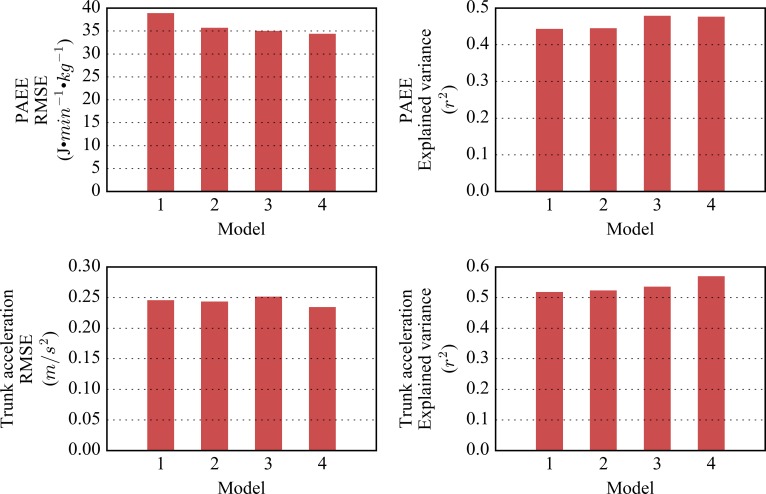
Performance of the four models of wrist acceleration. Explained variance shown is between-individual explained variance from ANOVA repeated measures.

The overall performance of each of the models in predicting both PAEE and trunk acceleration are shown in Fig 2. Between-individual explained variance in trunk acceleration was between 51% and 56% for all models. For PAEE, there were only minor differences between models in terms of explained variance, ranging from 44% to 47%; but there were slightly more pronounced differences in RMSE, ranging from to 38.8 J•min^-1^•kg^-1^ at worst to 34.4 at best. (For reference, 1 standard MET is 71 J•min^-1^•kg^-1^.) Model 4 was the strongest model to discriminate activity intensity levels, as it yielded the lowest RMSE for both criterion measures. The derived PAEE and trunk acceleration equations for each model are listed in the appendix (S1 Table and S2 Table).

Model 1 contains a linear term for ENMO, which is the most common signal derivative in current use for wrist acceleration data; it explained 44% of the between-individual variance in PAEE and has a RMSE of just above 0.5 METs.

The family of models using HPFVM as the wrist acceleration metric generally outperformed their ENMO counterparts by 2 to 3% in predicting both trunk acceleration and PAEE. The quadratic models outperformed their linear counterparts, decreasing RMSE by 2 to 8% implying that the relationships between wrist acceleration and both trunk acceleration and PAEE are curvilinear, rather than linear.

**Fig 3:**
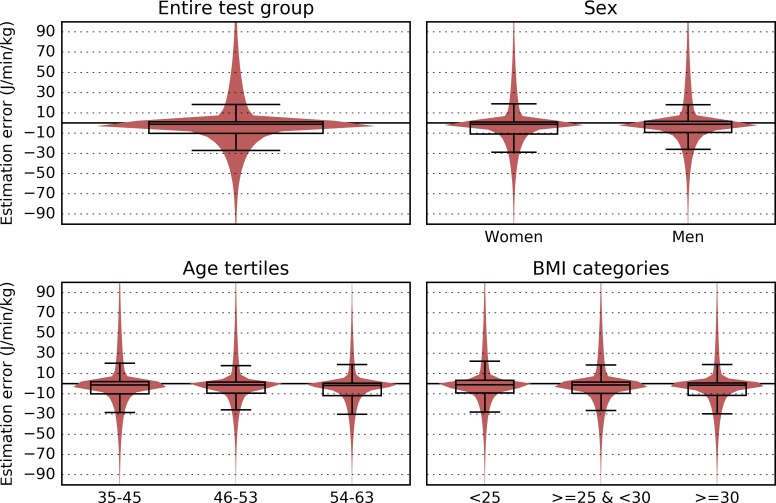
Violin plots and boxplots showing the estimation bias of model 4 across the whole test group (top left), by sex (top right), by age tertiles (bottom left) and BMI categories (bottom right).

Comparing the predictions of model 4 to PAEE from combined sensing in the cross-validation sample (n = 645) showed a negligible mean bias (0.07) with 95% limits of agreement between -70.6 and 70.7 J•min^-1^•kg^-1^ (Fig 3, panel 1). Stratified by sex, results indicated a 1.2 J•min^-1^•kg^-1^ overestimation in women, and a 1.8 J•min^-1^•kg^-1^ underestimation in men. Age and BMI were centred on their means for this analysis, therefore their coefficients imply a trend from underestimation in the younger and less obese towards overestimation in the older and more obese (0.2 J•min^-1^•kg^-1^ per year relative to mean age, and 0.3 J•min^-1^•kg^-1^ per kg/m^2^ relative to mean BMI). The distribution of this estimation error is visualised in Fig 3 using violin plots and overlaying traditional boxplots; the first panel shows the error distribution in the whole test set, and the remaining panels show error distributions within specific groups within the test set for comparison. It can be seen that estimation error was densely concentrated around zero for all groups, that there were no unusual estimation artefacts, and there were no outstanding differences between any of the groups.

**Fig 4:**
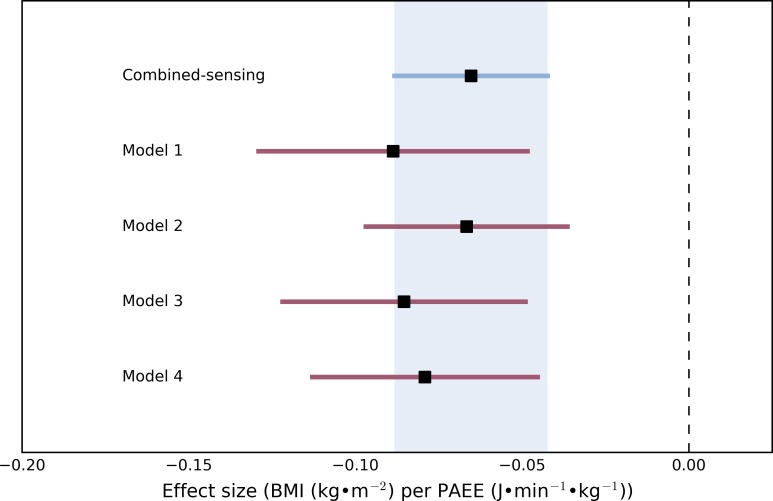
Forest plot showing the beta point estimates and their respective confidence intervals of the effect size of PAEE on BMI.

The association between PAEE and BMI was inverse across all models; the beta coefficients and their respective confidence intervals are visualised in the forest plot in Fig 4. All but one of the point estimates from the prediction models fell within the 95% confidence interval of the combined-sensing beta coefficient, and all confidence intervals from the wrist models overlapped the point estimate from combined sensing. The one outlying point estimate was from model 1, the weakest performing model according to other evaluations; however its quadratic counterpart (model 2) yielded the closest matching beta coefficient of all models.

## Discussion

To our knowledge, this is the first study to describe the validity of predicting high-resolution free-living PAEE from wrist acceleration in a large population sample of adult men and women, allowing evaluation of model performance in population subgroups.

Simple models of wrist acceleration intensity were found to explain a high proportion of variance in both PAEE and trunk acceleration, with no evidence of significant difference in bias by age or BMI categories but small opposite biases in men and women (underestimation and overestimation of PAEE, respectively).

The derived equations with non-linear terms were not monotonic; however the non-linear terms responsible for these were statistically significant in all cases. The global maxima of these equations (983mg and 1369mg in models 2 and 4, respectively) are likely reflective of the highest observed activity levels within the measured population; the data is naturally densely concentrated in the low end of physical activity intensity, and very sparse at the high end, therefore the downward trend at the high end can be considered an artefact of overfitting to the lower end. In practice, an implementation of these equations should truncate the estimates to the global maxima and minima (or zero) where appropriate.

The slightly better performance of HPFVM models compared to ENMO models suggests that applying a high-pass filter to the VM signal may be a more effective approach to the removal of gravity from an acceleration trace. However, the result of this filtering is likely to be dependent upon various signal properties, such as the machine noise level and sampling frequency, and the rotational frequency of human movement with respect to gravity [20].

A traditional validation study would only be able to report the estimation error structure, leaving readers to speculate whether similar associations between a measured behaviour and an outcome would be observed, irrespective of method. We compared the associations between PAEE and BMI as an example; the beta coefficients in the models for predicted PAEE were strikingly close to the beta-coefficient for PAEE from combined sensing, with a strong overlap of the 95% confidence intervals. This final analysis demonstrates that a similar direction and magnitude of relationship between PAEE and BMI can be observed in this population, regardless of whether PAEE is estimated by wrist acceleration or combined-sensing.

The models explored in this analysis only utilised the magnitude of wrist acceleration for prediction, and still achieved strong results. There is potentially a greater explanatory power to be found in the multitude of signal features that are derivable from three-dimensional acceleration in waveform resolution. Nonetheless, we should be cautiously optimistic that even the basic and robust properties of this easily obtainable and commonly used measure are strongly related to the criterion measure of PAEE from combined sensing.

The validity of these analyses is naturally contingent upon the validity of the criterion measure, individually-calibrated combined sensing of heart rate and trunk acceleration. While it is not considered a gold-standard measurement of PAEE, this estimation method does have established validity of both intensity [13,14] and PAEE during free-living in the population used for the present evaluation [18], and to our knowledge this study currently represents the largest aggregation of simultaneous wrist acceleration and energy expenditure signals in free-living.

An additional potential limitation of this study is that it is neither nationally or globally representative, but confined to a relatively affluent and culturally homogenous population living in the East of England. The prevalence of many activities, during which wrist acceleration may be more or less representative of PAEE, is likely determined by several factors such as culture, climate, and local landscape, and it is therefore possible that the specific relationships and error structures that we report here may not be universal. Still, our analytical sample comprises both men and women across a wide BMI and activity level range, thus providing a comparative framework for interpreting wrist acceleration data from population studies.

In conclusion, we have demonstrated that a strong relationship exists between PAEE and wrist acceleration. The best performing model explained 47% of the between-individual variance in PAEE with a RMSE of 34 J•min^-1^•kg^-1^ (0.48 METs) and all prediction models produced similar associations with BMI. Further work should aim to improve upon the accuracy of PAEE prediction using a wider range of the signal feature space, and to explore generalizability in other populations.

## Supporting Information

S1 Table Derived regression models of PAEE. (DOCX)

S2 Table Derived regression models of trunk acceleration. (DOCX)
