# Supplementary material for: Estimation of Physical Activity Energy Expenditure during Free-Living from Wrist Accelerometry in UK Adults
Source: PLoS One. 2016 Dec 9;11(12):e0167472. doi: 10.1371/journal.pone.0167472 (PMC5147924; doi:10.1371/journal.pone.0167472)
Supplement: S1 Table — (DOCX) [file pone.0167472.s001.docx]

| Model | Formula to predict PAEE (J•min^-1^•kg^-1^) | Within-individual r^2^ | Between-individual r^2^ | RMSE (J•min^-1^•kg^-1^) |
| --- | --- | --- | --- | --- |
| 1 | $5.01 + 1.000\times ENMO$ | 0.60 | 0.44 | 38.8 |
| 2 | $-10.58+1.1176\times ENMO+2.9418\times\sqrt{ENMO}-0.00059277\times{ENMO}^{2}$ | 0.66 | 0.44 | 35.7 |
| 3 | $-4.65+ 0.8537\times HPFVM$ | 0.68 | 0.47 | 35.0 |
| 4 | $-1.25+1.1353\times HPFVM -2.4281\times\sqrt{HPFVM}-0.00040270\times{HPFVM}^{2}$ | 0.69 | 0.47 | 34.4 |

S1 Table. Derived regression models of PAEE.
