# Supplementary material for: Estimation of Physical Activity Energy Expenditure during Free-Living from Wrist Accelerometry in UK Adults
Source: PLoS One. 2016 Dec 9;11(12):e0167472. doi: 10.1371/journal.pone.0167472 (PMC5147924; doi:10.1371/journal.pone.0167472)
Supplement: S2 Table — (DOCX) [file pone.0167472.s002.docx]

| Model | Formula to predict trunk acceleration (m•s-^2^) | Within-individual r^2^ | Between-individual r^2^ | RMSE (m•s-^2^) |
| --- | --- | --- | --- | --- |
| 1 | $-0.057 + 0.0060321\times ENMO$ | 0.59 | 0.51 | 0.245 |
| 2 | $0.0423+0.0087\times ENMO-0.03860\times\sqrt{ENMO}-0.00000129\times{ENMO}^{2}$ | 0.59 | 0.52 | 0.243 |
| 3 | $-0.097+ 0.0047835\times HPFVM$ | 0.57 | 0.53 | 0.251 |
| 4 | $0 .114+0.007367\times HPFVM -0.057613\times\sqrt{HPFVM}-0.000001428\times{HPFVM}^{2}$ | 0.62 | 0.56 | 0.234 |

S2 Table. Derived regression models of trunk acceleration
